# Perceptions of cervical cancer prevention on Twitter uncovered by different sampling strategies

**DOI:** 10.1371/journal.pone.0211931

**Published:** 2019-02-11

**Authors:** Gem M. Le, Kate Radcliffe, Courtney Lyles, Helena C. Lyson, Byron Wallace, George Sawaya, Rena Pasick, Damon Centola, Urmimala Sarkar

**Affiliations:** 1 UCSF Center for Vulnerable Populations, Division of General Internal Medicine, Department of Medicine, UCSF, San Francisco, CA, United States of America; 2 College of Computer and Information Science, Northeastern University, Boston, MA, United States of America; 3 Department of Obstetrics and Gynecology, UCSF, San Francisco, CA, United States of America; 4 Helen Diller Family Comprehensive Cancer Center, UCSF, San Francisco, CA, United States of America; 5 Annenberg School of Communications, University of Pennsylvania, Philadelphia, PA, United States of America; University of Zurich, SWITZERLAND

## Abstract

**Introduction:**

Cervical cancer prevention is possible through use of the HPV vaccine and Pap tests, yet the vaccine remains underutilized.

**Methods:**

We obtained publicly-available Twitter data from 2014 using three sampling strategies (top-ranked, simple random sample, and topic model) based on key words related to cervical cancer prevention. We conducted a content analysis of 100 tweets from each of the three samples and examined the extent to which the narratives and frequency of themes differed across samples.

**Results:**

Advocacy-related tweets constituted the most prevalent theme to emerge across all three sample types, and were most frequently found in the top-ranked sample. A random sample detected the same themes as topic modeling, but the relative frequency of themes identified from topic modeling fell in-between top-ranked and random samples.

**Discussion:**

Variations in themes uncovered by different sampling methods suggest it is useful to qualitatively assess the relative frequency of themes to better understand the breadth and depth of social media conversations about health.

**Conclusions:**

Future studies using social media data should consider sampling methods to uncover a wider breadth of conversations about health on social media.

## Introduction

Despite being highly preventable and treatable, cervical cancer continues to cause significant morbidity and mortality in young to middle-aged women.[1] Cervical cancer is preventable with the HPV vaccine, and early detection of cervical cancer through Pap testing prevents morbidity and mortality.[2] However, HPV vaccination rates in the United States (US) are sub-optimal with only 60.4% of teens ages 13 to 17 years old ever initiating the vaccine and 43.4% completing the vaccine, which are well below the Healthy People 2020 goal of 80%.[3] Increasing the acceptance and uptake of the HPV vaccine is expected to have critical impact in closing the gap in prevention behavior and reducing incidence of cervical and other HPV-related cancers.

Social media plays an important role in health communication and may promote uptake of HPV vaccination and Pap testing. Social media channels have transformed the ways that society shares ideas, beliefs, news, and information[4] about products and services among individuals and organizations.[5] Online social networks such as Twitter and Facebook have been an effective platform for disseminating and counteracting health messages, particularly in young adults.[6,7] In 2015, 67% of US adults and 90% of those age 18 to 29 used social media, with high usage reported across all racial/ethnic groups.[8]

A critical concern in using online social networks for public health promotion is understanding the social media dialogue about health behaviors. Automated approaches can include more data than traditional qualitative analyses, but the complexity of user-generated social media content does not lend itself to meaningful interpretation without expert human analysis.[9,10] For example, a recent methodological study showed that traditional qualitative analyses are needed to provide more nuanced and important details to provide context for themes in social media data.[10] Guetermann et al compared traditional qualitative analysis with an automated approach (natural language processing, NLP) augmented by qualitative analysis and found that the augmented approach was an efficient methodology that was able to capture most themes. However, the authors determined that the automated approach alone lacked the ability to detect nuances for more meaningful interpretations of the data. This combined approach has been further supported as a useful research framework in a recent narrative review of 18 studies that analyzed Twitter data in health care research.[10] In their narrative review, Hamad and colleagues identified the key features of these studies and used them to propose a combined content analysis model for analyzing social media data. They argue that the advancement of social media research would benefit from a combined approach of automated and manual approaches to content analysis.

Therefore, we sought to combine automated analysis and qualitative coding, following best-practices for mixed-methods research[5] to characterize the ways in which users use Twitter to discuss cervical cancer prevention and detection. Our primary goal was to conduct a rigorous, descriptive content analysis of tweets derived from different sampling strategies. A secondary objective of this study was to provide insight on how common sampling strategies of Twitter data may yield different themes in qualitative analyses.

## Methods

### Data source

**Fig 1:**
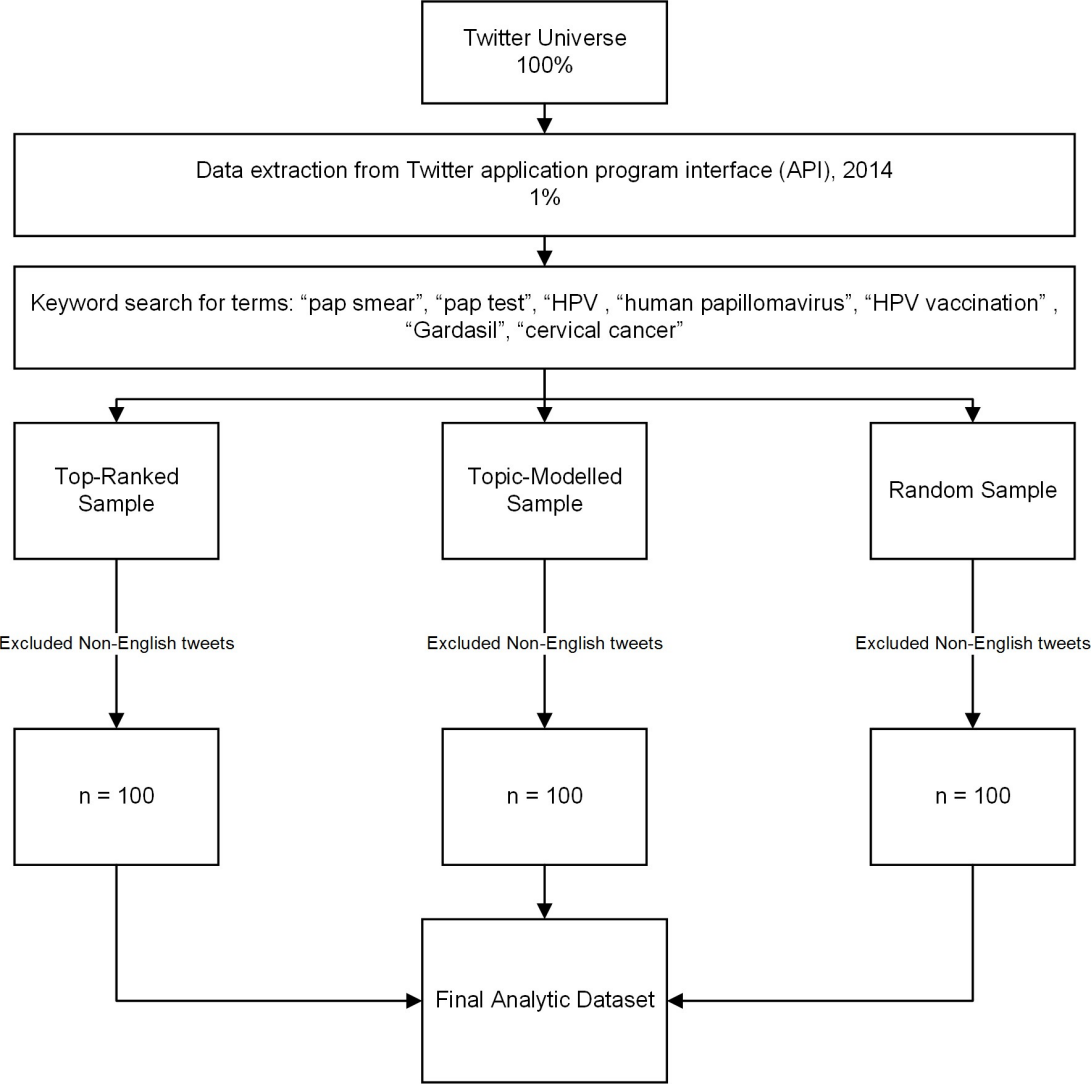
Study flow chart.

Twitter is a widely used micro-blogging website that allows users to post public messages called “tweets” limited to 280 characters (www.twitter.com). Tweets may contain links to other content, such as images, websites or news articles. We accessed a free 1% sample of tweets from Twitter’s publicly-available application program interface (API) extracted during 2014 over the period of March 2012 –March 2014 (Fig 1). We then searched for messages relevant to cervical cancer screening and prevention by selecting tweets including the following search terms and hashtags: “pap smear”, “pap test”, “HPV, “human papillomavirus”, “HPV vaccination”, “Gardasil” (trade name for a common HPV vaccine), “cervical cancer”.

We de-identified messages by removing the username from tweets presented in this paper; if usernames are mentioned in the tweets, we denote this by @XXXX. Our sample was limited to English-language messages only. This study was approved by the Insitutional Review Boards at UCSF and University of Pennsylvania. This study did not require participant consent for the use of publicly available data. We have complied with the Terms of Service for the use of Twitter data (https://twitter.com/en/tos, 12/12/2018).

### Sampling methodologies

We purposively sampled the social media content from the large Twitter corpus to identify the most common themes and describe less common themes and outliers to delineate the full range of responses. We aimed to capture these themes in our content analysis using three sampling strategies: 1) the top-ranked (most-retweeted) tweets, 2) simple random sample of tweets, and 3) a standard statistical “topic modeling” approach using Latent Dirichlet Allocation, or LDA.[11]

Topic modeling refers to a family of statistical models that aim to uncover latent thematic structure in texts. Such models assume that ‘topics’ may be viewed as conditional word probabilities, and that documents (in our case, tweets) contain a mixture of topics, which in turn give rise to the words that they comprise. LDA is the most common variant of topic modeling, and the one used in the present work. This model draws mixtures over topics in a document from a Dirichlet distribution, hence its name. A specific word *w* in a given document is then assumed to be drawn from a particular topic *z*, which was in turn selected with probability proportional to the mixture weights drawn from the Dirichlet distribution governing topic mixture parameters. We used the implementation of LDA available in the Gensim Python package (version 12.2.0).[12] We assumed 20 topics and used a symmetric α hyperparameter for the Dirichlet distribution of 0.001. From these topics, we included 100 tweets across the 20 topics (the first 5 tweets from each topic) in our topic model sample.

The number of tweets that we used in our content analysis depended on the distribution of results. We extracted data iteratively using the top-ranked, random-sampling and topic-modeled approach until we reached thematic saturation[13] to ensure comparable numbers across sample sizes for meaningful frequency comparisons. The analytic dataset thereby contained a sample of 300 tweets from the keyword results, with 100 tweets determined as point of reaching thematic saturation for each of the three sampling methods.

### Coding

Two members of the research team (GL, KR) applied descriptive content analysis[14] to the cervical cancer social media content from these searches using Dedoose[15] (www.dedoose.com) to capture the range of themes. Dedoose is a cost-efficient web-based application, which was preferred over other platforms for its user interface and efficiency for mixed methods research. We qualitatively coded tweets based on: 1) content of the tweet, 2) URLs and images embedded in tweets, 3) “activity” around the original tweets (likes, retweets, conversations), 4) user profile of the tweeter, and 5) sources of information for tweets that included links (e.g., internet news sources). Based on the user profile, we determined whether a message was from an individual or an organization by coding the “About Me” section of the page. We further coded whether the tweet was generated by a verified user, which is denoted by a blue badge next to the user name and indicates that the Twitter account of public interest is authentic, particularly for individuals and organizations in key interest areas such as music, acting, fashion, government, politics, religion, journalism, media, sports, and business. We focused on these two key profile characteristics of Twitter users in our analysis because they have been identified as influential factors in predicting the number of followers,[11] and thereby increasing the likelihood that a message is shared.[12,16]

### Analysis

Our content analysis of the messages was informed by grounded theory,[5,17] allowing categories to emerge inductively as the coding progressed. Two research team members read a sample of the messages and generated an initial list of “observer identified” themes[18] which were then developed into a coding template or codebook by consensus. These categories (codes) were entered into a qualitative data analysis platform[12] and applied to the remaining social media messages; we also employed open-coding and re-coding to identify novel themes that the team discussed during analysis meetings. Once all of the textual data had been coded, the research team (GL, KR, HCL) re-read the messages within themes and developed analytical sub-categories to understand underlying reasons for more granular themes (e.g., underlying reasons for Pap test discomfort). All categories identified were discussed in a consensus process, and open-coding categories were added to the codebook during analyses. The analysis presented in this paper is organized as follows: 1) an in-depth narrative discussion of overall themes that emerged from the combined data and 2) a comparison of themes and their frequency across sample type and user type.

## Results

### Tranversal themes from the data

We identified four prevalent themes that emerged from our qualitative analysis of tweets that cut across all sample types: Theme 1) Cervical cancer prevention discussed on social media uses positive messaging to encourage low-effort/ high-reward behavior and female empowerment, Theme 2) “Pap smear” is used as a stigmatized term, but there is an ongoing effort to de-stigmatize the term on social media, Theme 3) Largely positive public opinion about HPV testing and vaccination, and Theme 4) Prevalent liberal political views on women’s rights, reproductive health, and access to care in the era of the Affordable Care Act. Taken together, these themes are centered on perceptions of cervical cancer prevention messages as they relate to decision-making, social norms, and political views. We discuss each theme below and highlight representative tweets as a window into public perceptions shared on social media.

#### Theme 1. Cervical cancer prevention discussed on social media uses positive messaging to encourage low-effort/ high-reward behavior and female empowerment

One major theme focused on the framing of cervical cancer screening promotion on social media in terms of the benefits of obtaining screening (framed as a “gain” message) rather than the cost of not obtaining screening (framed as a “loss” message). Cervical cancer prevention messages were framed positively and focused on the benefits of screening:

“Thanks to #exposepp a Pap smear in college (no $) saved my life which allowed me the choice of 2 beautiful children today. PPFamilyValues” (unverified individual, topic-modeled sample)

Loss-framed messages that highlighted the harms of lack of screening were not as commonly found on Twitter:

“12 women die of cervical cancer everyday!12! Imagine that?be INFORMED.Tell a friend,sister,daughter,mother and all follow @BraveheartsPhil” (unverified individual, topic-modeled sample)

We also observed that Twitter discussion about cervical cancer was largely focused on the message of prevention; the term “prevent” (or variations) was found in the text or hashtags of 13% of tweets in the random sample, 7% in the topic-modelled sample, and 14% in the top-ranked sample:

“#PreventCervicalCancer Go for a Pap smear test regularly..Pap smear tests are free at all Botswana public hospitals,” (unverified individual, topic-modeled sample)

Not only was prevention a major focus of tweets, these messages showed a persuasive tone by framing cervical cancer prevention as easy to accomplish and addressed barriers to obtaining screening. For example, in the random sample, 10% of tweets promoted this message through various strategies, including use of terms related to costs, specific instructions related to how to obtain screening, and information about new prevention strategies[10,16]:

“Join Shimmers Preventive Movement against Cervical Cancer.we organise fun parties,provide screenings @ d venue.results r given on d spot.” (unverified individual, random sample)

“Approx. 900 cervical cancer cases are diagnosed a year in NY. Get screened for one of the most preventable cancers” (verified individual, random sample)).

Tweets discussing cervical cancer prevention contained positive language that informed followers of the ease and affordability of prevention. Both high-tech and low-tech strategies were highlighted as potential strategies for early detection of cervical cancer.

“Smartphones are capable of detecting everything from an ear infection to cervical cancer: http://t.co/4PP3m53qXy *http://t.co/WWc3gpHA3B*” (verified organization, random sample)

“Vinegar Test Accurately And Cheaply Screens For Cervical Cancer *http://t.co/zihYle8QCc*” (unverified organization, random sample)

Tweets about cervical cancer prevention commonly contained messaging framed as advocacy for women’s health. Across all samples, 12–20% of tweets used language such as “we” (rather than “you”), “every woman,” and “ladies,” which may contribute to a sense of female solidarity and togetherness in prevention through empowerment:

“#LadiesOughtTo go for Pap smear..extremely important for early detection of Cervical Cancer risks …pls think abt it.” (unverified individual, random sample)

“As part of Cervical Cancer Prevention Week, we encourage you to get checked #SmearforSmear #CCPW *http://t.co/ncV0nhVVUs”* (verified organization, random sample)

#### Theme 2: “Pap smear” is used as a stigmatized term, but there is an ongoing effort to de-stigmatize the term on social media

Another prevalent theme that emerged was the manner in which the term Pap smear/ test was used by Twitter users. The term Pap smear/ test was referenced in 34–41% of tweets across sample types. However, the manner in which the term was used varied widely, from a positive means to raise awareness of cervical cancer prevention to a crude joke unrelated to cervical cancer or embedded within political commentary. The heterogeneous use of the term on Twitter reveals a discernable tension between references that reinforce the stigmatic and negative associations with the procedure, versus attempts to de-stigmatize pap smears and promote them as an effective cancer prevention procedure:

“Cancer screening is more than mammograms. It's also pap smears, colposcopy & breast exams. *#PinkOut”* (unverified organization, top-ranked sample)

“Morning! Next week is Cervical Cancer Prevention Week–make sure you have your smear *#CCPW* x x x” (verified individual, top-ranked sample)

Tweets that referenced pap smears in a positive way tended to focus on alerting women to the importance of getting pap smears to prevent cervical cancer. In the top-ranked sample, these tweets came from a mix of verified (n = 8) and unverified (n = 6) users, with individual users (n = 10) sharing these positive messages surrounding pap smears more than organizations (n = 4). For example:

Nairobi Womens Hospital are offering free breast cancer and Pap smear every Saturday this month. Share widely! (verified individual, top-ranked sample)

In addition, attempts to destigmatize the term “Pap smear” were prominent only in the top-ranked sample through the use of the hashtag #SmearForSmear, a social medial campaign developed by Jo’s Trust, a UK cervical cancer charity. The #SmearforSmear campaign encouraged individuals to use the hashtag to post a photo of themselves on social media with lipstick smeared on their face, and to nominate a friend to do the same. For example:

“As part of Cervical Cancer Prevention Week, we encourage you to get checked *#SmearforSmear#CCPW*” (verified organization, top-ranked sample)

“*#SMEARFORSMEAR* raise awareness for cervical cancer and post a smear selfie … While you're at it..… *http://instagram.com/p/ykl126Ix1C/”* (verified individual, top-ranked sample)

“Cervical cancer campaign *#smear4smear* I nominate *@elizhilfigz* @missjourdandunn elenaora… *http://instagram.com/p/yY-4Twxs4c/”* (verified individual, top-ranked sample)

By relating the often-stigmatized pap smear procedure to smearing lipstick on one’s face, this social media campaign represents an important effort by a cancer organization to destigmatize pap smears, which gained traction among influential users, such as verified users in the top-ranked sample.

Furthermore, that four of the five users that tweeted the #SmearForSmear hashtag in the most retweeted sample were verified users demonstrates the ability of a positive, cancer prevention message to gain traction among highly visible, influential, and reputable users on Twitter.

While organizations like Jo’s Trust have deliberately embraced the use of the term pap smear as a way to raise awareness of cervical cancer screening on social media, the use of the term by other users on Twitter reinforces the stigma and negativity often associated with the procedure in mainstream culture. In particular, many tweets that referenced pap smears in a negative way decontextualized the procedure from a women’s health context and associated it with a crude insult or joke. Individual and unverified users were most likely to share tweets that reinforced the stigma of Pap smears by using the term negatively:

“When the paparazzi releases an unflattering article about someone, is it called a pap smear?” (verified individual, top-ranked sample)

“Would rather get a pap smear with a rake than work on Mondays” (top-ranked sample, unverified individual)

#### Theme 3: Largely positive public opinion about HPV testing and vaccination

Given the recent change in cervical cancer screening guidelines to include HPV testing and vaccination, we expected to see some discussion on Twitter on this topic. We found that there was a low frequency of tweets expressing an opinion about HPV testing and vaccination across all samples, but the content of the tweets shared on Twitter were overall positive and encouraging. Tweets expressing sentiment, positive or negative, about the HPV vaccine were relatively infrequent across all samples (10% of tweets in the topic-modelled sample, 6% in the top-ranked sample, 8% in the random sample), however vaccine-related messages primarily promoted its use as a safe and effective means of preventing cervical cancer with large potential to eradicate the burden of the disease on a global scale:

“HPV vaccine can help prevent cervical cancer. Why aren't more girls getting vaccinated? Read our Expert Voices blog: *http://t.co/ig9Z05z3xr*” (verified organization, topic-modeled sample)

“If pre-teen & teen girls receive the #HPV vaccine, they can avoid cervical cancer. Yet, uptake is stalling in the US: *http://t.co/tnSAcM74QS*” (unverified individual, topic-modeled sample)

We also found that sample tweets often provided links to peer-reviewed scientific literature as further support for positive vaccine sentiment.

“HPV shots don't make girls promiscuous, study says: Shots that protect against cervical cancer do not make girls … *http://t.co/z4plODOQ”* (unverified organization, topic-modeled sample)

“HPV vaccination could reduce global deaths from cervical cancer by two-thirds http://t.co/KyMlOhkYrw #WorldCancerDay *http://t.co/4dKTOIW964”* (verified organization, topic-modeled sample)

Although the topic-modeled and top-ranked tweet samples did not contain any examples of anti-HPV vaccine sentiment, the study team coded replies to sample tweets, which contain examples of anti-vaccine sentiment on Twitter. The examples below indicate the sample tweet included in analysis as well as the anti-vaccine reply to the tweet:

Sample tweet: “May is Cervical Cancer awareness month! Get your Cervarix shot today and protect yourself for life #xmeanslove _ *http://t.co/761YpuTbBd”* (unverified individual, top-ranked sample)

Anti-vaccine response: “139 #girls have died from #HPV #vaccinations http://www.trueactivist.com/its-official-139-girls-have-died-from-hpv-vaccinations/ … #Gardasil” (unverified individual, top-ranked sample)

Sample tweet: “Scientists find no serious side effects to HPV vaccine. Scientists kicking cervical cancers ass: 1 Wannabe smart person @XXXX: 0” (verified individual, top-ranked sample)

Anti-vaccine response: “@XXXX stats of vaccine related injuries&death for this are truly frightening. Regular GYN care is key for detection and prevention.” (unverified individual, top-ranked sample)

Within the random sample, tweets with anti-vaccine sentiment use similar language to the replies to pro-vaccine tweets, primarily expressing concern about vaccine safety.

“@TalkIBC @tuscaloosanews 1:40K get cervical cancer,yet Gardacil causes over 100 deaths year& other major complications;is Gardacil about $ $” (unverified individual, random sample)

#### Theme 4. Prevalent liberal political views on women’s rights, reproductive health, and access to care in the era of the Affordable Care Act

A major theme that emerged was the political nature of the tweets as they related to women’s reproductive health rights and access to health care. Because cervical cancer is associated with reproductive health, the cancer screening discussion on Twitter is often bundled with other women’s health issues, such as mammograms and prenatal care:

“45M women have already utilized services like mammograms, pap smears, and prenatal care at no cost thanks to Obamacare. *http://t.co/LX57y7H9”* (unverified organization, topic-modeled sample)

“Planned Parenthood provides 500k breast exams, 400k cervical cancer screenings per year#StandWithPP to protect their essential services.” (verified individual, topic-modeled sample sample)

A majority of these political tweets expressed a liberal perspective on women’s health and support of Planned Parenthood (PP). For example:

“BREAKING: House GOP votes to defund Planned Parenthood, replace all those pap smears and breast exams for poor women with prayer” (unverified individual, top-ranked sample)

“Women taking birth control and getting Pap smears are not a threat to national security” (unverified individual, top-ranked sample)

Among the tweets that mentioned Pap smears as part of a political commentary across all samples, a majority came from individual and/ or unverified users on Twitter. Many tweets were directed to political parties and individual politicians:

“@GovHerbert I'll come right to you for my PAP smear since you defunded the only preventative healthcare provider I can afford as a student” (individual, random sample)

Because the study period (2014) covered the early era of the Affordable Care Act (passed in 2012, enacted in 2014), several tweets expressed strong opinions from both sides of the political spectrum, expressing support (“*45M women have already utilized services like mammograms*, *pap smears*, *and prenatal care at no cost thanks to Obamacare*. *http://t.co/LX57y7H9*”) or opposition (“*Today was #myfirsttime I was denied a pap smear b/c Obamacare now requires 1 every 3 years instead of annually*. *Who’s waging a war on women*?*”*). A minority of these political tweets presented a critical perspective of the Affordable Care Act or of Planned Parenthood:

“Women's health outcomes will worsen with the new Pap smear rules and shift of Manmograms to 40 and up. Stop lying @BarackObama” (unverified individual, random sample)

“Look, ladies, if mammograms and pap smears and these things Planned Parenthood does are so important, then why don’t men need them?” (unverified individual, top-ranked sample)

Of the tweets that mentioned pap smears as part of a political commentary across all samples, a majority came from users who were individuals and/ or unverified users on Twitter.

### Comparison of themes across three sampling methodologies (Fig 2 and Table 1)

**Fig 2:**
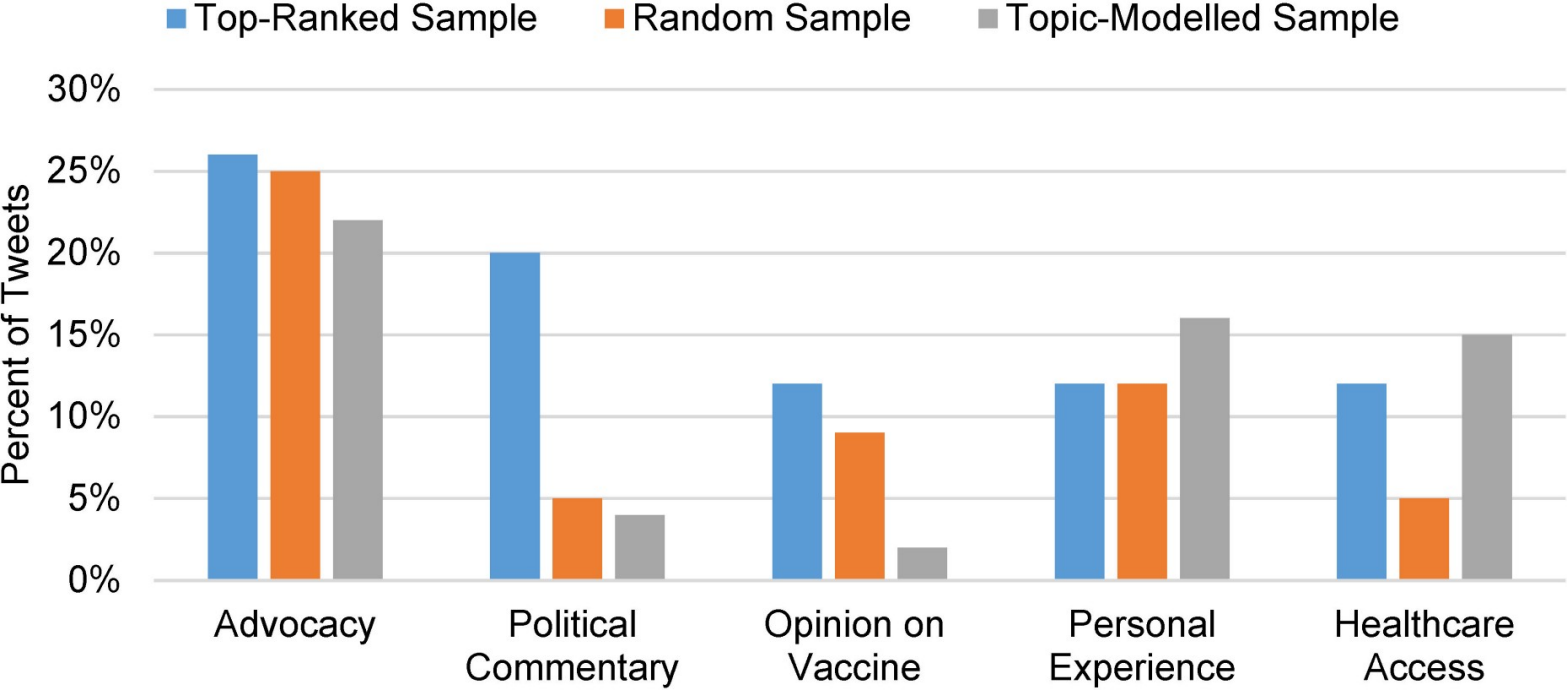
Key theme domains by sample type.

Fig 2 shows the occurrence of themes that emerged across the three different sample types: 1) political commentary: tweets that reference political figures or policy, 2) opinion on vaccine: tweets with a subjective perspective in support or opposition of the HPV vaccine, 3) advocacy: tweets that advocate or advise followers to take an action in prevention of cervical cancer, or that describe prevention efforts, 4) personal experience: tweets that share an individual’s experience with cervical cancer or HPV, either from the individual or a surrogate communicator, and 5) healthcare access: tweets that discuss barriers to obtaining healthcare, including the cost of care. Advocacy-related tweets constituted the most prevalent theme to emerge across all three sample types, and were most frequently found in the top-ranked sample. Tweets related to a political theme were more common in the top-ranked sample and rarely appeared in the random and topic-modelled samples. Similarly, opinions about the HPV vaccine were more commonly found in the top-ranked and random sample, and tweets expressing these opinions were least likely to be represented in the topic-modelled sample. Tweets related to personal experience and health care access were most commonly found in the topic-modelled sample; however, personal experiences were equally represented in both the top-ranked and random sample. Tweets related to health care access were less common in the random sample. Table 1 shows example tweets for each theme across all three sample types.

**Table 1:** Themes and example tweets by sample type.

Theme	Definition	Top-Ranked Example Tweets	Random Sample Example Tweets	Topic Model Example Tweets
Advocacy	Tweets that advocate or advise followers to take an action in prevention cervical cancer, or that describe prevention efforts	Guys join me and help raise awareness for cervical cancer this jan. Get screened FREE at GKLF 1a Unity rd off … http://t.co/bbTzvgq0	Approx. 900 cervical cancer cases are diagnosed a year in NY. Get screened for one of the most preventable cancers. http://t.co/GaLMeWg20s	#PreventCervicalCancer Go for a Pap smear test regularly..Pap smear tests are free at all Botswana public
Political commentary	Tweets that make reference to political figures or policy.	Also gone if Romney repeals Obamacare: full coverage of preventative care, like contraception, mammograms, pap smears. #debate	Pants on Fire! @TXDemParty says if #HB2 passes, El Pasoans must drive to San Antonio for cervical cancer tests http://t.co/CQtrimMAB6	Should I vote 4 Bernie Sanders that thinks orgasms prevent cervical cancer or the one who has an illegitimate child? http://t.co/LDCbzXqrQq
Opinion on vaccine	Tweets that share a subjective perspective in support or opposition of the HPV vaccine	PLS GET YOUR KIDS VACCINATED SO THEY DONT GET CERVICAL CANCER OR GIVE HPV TO SOMEONE. U THINK PPL ENJOYED POLIO? IT'S 2014. FKN ACT LIKE IT.	There will be 53,000 excess cervical cancers among girls who should be getting HPV vax bc of poor uptake in US, bad journalism = collateral damage	HPV vaccine can help prevent cervical cancer. Why aren't more girls getting vaccinated? Read our Expert Voices blog: http://t.co/ig9Z05z3xr
Personal experience	Tweets that share an individual’s experience with cervical cancer or HPV, either from the individual or a surrogate communicator	@XXXX I'm 35 and going thru cervical cancer. Plz help me RT reminder for all women to go for their annual check ups! So important!	@XXXX sadly a friend19 died of cervical cancer Saturday after being refused a smear test. Help spread the word http://t.co/cE26HeHapv	Thanks to #exposepp a Pap smear in college (no $) saved my life which allowed me the choice of 2 beautiful children today. PPFamilyValues
Healthcare access	Tweets that discuss barriers to obtaining healthcare, including mentioning the cost of care	Thanks to #PlannedParenthood (one of MANY reasons) dirt-poor 19 yo me was able to find out I didn't have cervical cancer after irregular pap	Free Pap smear test till Friday in participating clinics! But most are fully booked. Try calling for an appt! http://t.co/hGzXxLjqOF	Don't have health insurance? Check out our Breast & Cervical Cancer Early Detection Program!

### Comparison of themes across user type (Fig 3)

**Fig 3:**
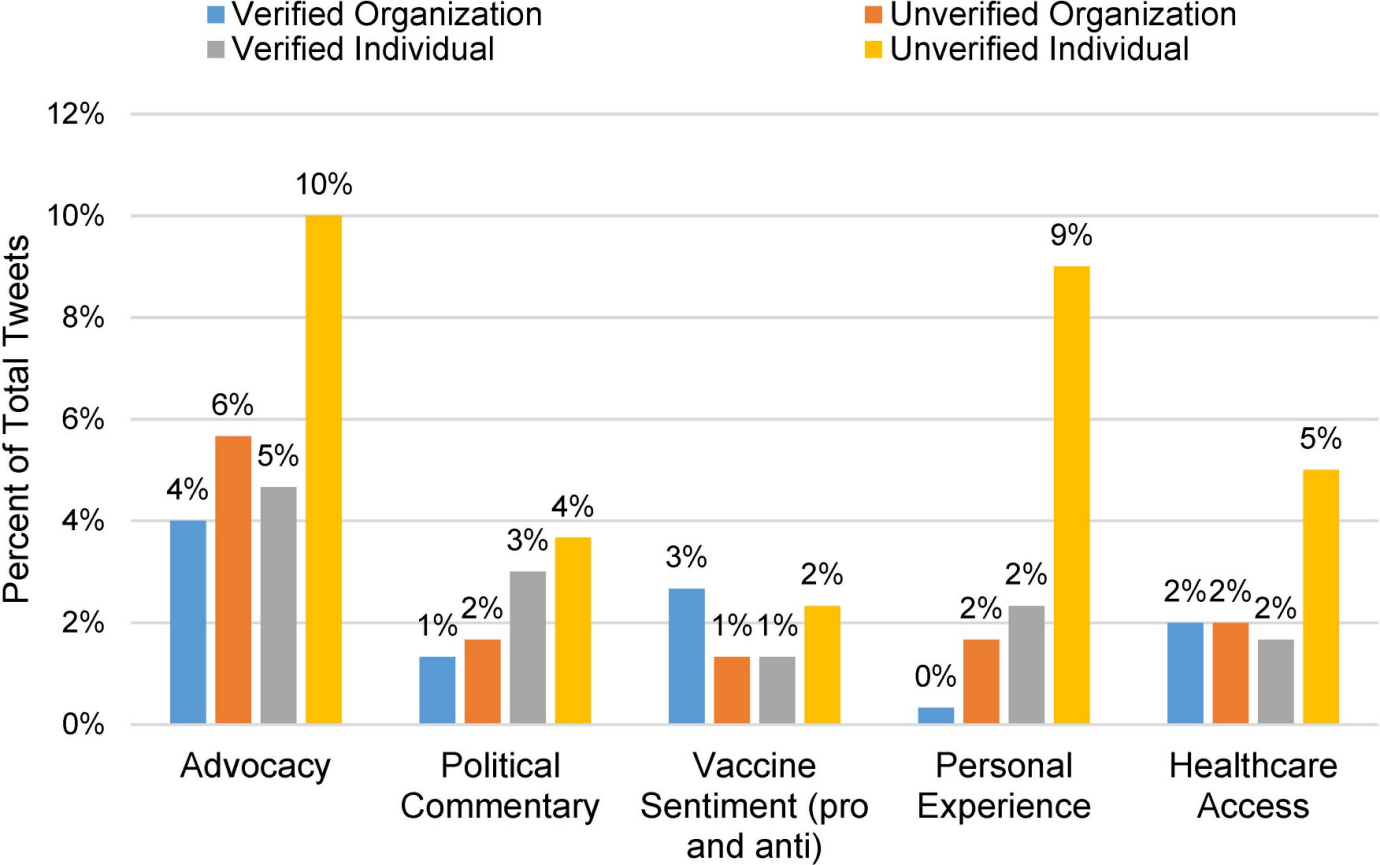
Key theme domains by user type.

Fig 3 shows the distribution of codes captured by user type: a) individual vs. organization and b) verified vs. unverified user. Overall, individual users were more likely to tweet than organizations across most themes, except for opinions about the HPV vaccine. Similarly, unverified users were more likely to tweet than verified users across all themes except for opinions about the HPV vaccine. Unverified users were more likely to sample tweet than verified users.

## Discussion

Studies using Twitter data are useful for characterizing how the public perceives health and how information spread through social media may affect health behavior[19]. Our study revealed valuable insights into the current nature and scope of cervical cancer prevention conversations on Twitter. The themes regarding cervical cancer prevention messaging are consistent with health communication research in other studies[20]^,^[21], in which the majority of themes have focused on prevention through screening and/or HPV vaccination, with most tweets expressing positive recommendations for uptaking cervical cancer prevention behaviors. However, our analysis deepens understanding in the current literature by contributing a more diverse and nuanced characterization of cervical cancer prevention discussions on Twitter, showing that specific themes vary in the intensity of appearance depending on the sampling methodology used.

We found that positive messaging emphasizing the benefits of adopting a specific health behavior rather than focusing on the negative harms of not adopting the behavior has been shown to be more effective strategy for framing health promotion messages.[22] The diverse ways in which pap smears were referenced across the samples holds important implications for the use of social media to generate positive public health messages and promote healthy behaviors. Specifically, this analysis reveals that positive messages pertaining to pap smears and cervical cancer prevention are more likely to be tweeted by verified users, while messages that reinforce the stigma associated with pap smears or express an overtly political opinion are more likely to come from unverified users. This underscores that verified users are crucial to facilitating the spread of positive, evidence-based public health messages on social media. It also suggests that there may be potential to address misinformation by identifying unverified users on Twitter and educating the public to be aware of the credibility of information tweeted by unverified users.

With regard to vaccination attitudes, prior studies have shown largely positive sentiment with regard to HPV vaccine on social media.[23]^,^[24]^,^[25] Prior studies have typically examined stand-alone tweets independently without incorporating replies to tweets in their content analysis[20]; our study provides a more nuanced landscape of the debates that occur within tweets about cervical cancer prevention, specifically with regard to the HPV vaccine, which has been subject of controversy among individuals who are hesitant about the vaccine. However, anti-vaccination messages that take place on Twitter in response to these positive sentiment tweets are important for understanding how to reach unvaccinated populations and engaging with them to dispel myths about the vaccine. For example, tweets expressing anti-vaccination sentiment show a mistrust of government sources of information or allude to a conspiracy theory with pharmaceutical corporations. Our findings indicate that while analyses may reveal a largely positive sentiment of the HPV vaccine on the surface, a more in-depth examination of the replies to the positive tweets about the vaccine show how anti-vaccine debates are active on Twitter and present an opportunity for public health advocates to engage in dispelling myths and correcting misinformation about the vaccine.

This study also demonstrated the ways in which sampling methods can affect the occurrence of themes in social media conversations, however, there has been little empirical work done regarding differences in these approaches and best practices for sampling and analyzing Twitter data. While a variety of methods have been used, there has been little guidance as to how these methods differ and the implications of these methods for interpreting data from social media data sources. Overall, our results showed that although the frequency of themes differed to some extent across sample types, the substance of the themes was prevalent in all samples. Specifically, we observed that the random sampling had the least political tweets and the most opinions on cervical cancer. It appeared that the topic model did not add substantively different themes over and above themes gleaned from analysis of random sampling. The random sample had the greatest breadth in topics, covering a wide range of content related to cervical cancer prevention. The top-ranked sampling appeared to show the greatest bias toward politicizing a public health topic.

Although small sample sizes may be a limitation for examining the frequency of the themes, thematic saturation within and across samples allowed us to determine that the sample size was sufficient to address the research question. Moreover, the strength of this study lies in its approach to examine several different sampling methods to draw potential inferences about how conversations about Twitter can be depicted differently depending on the sampling method used.

## Conclusions

This study showed that tweets with positive messages lend themselves to wider promotion and increased sharing through retweets, demonstrating the importance of this language for public health promotion. Researchers should not confine themselves to highly shared tweets in order to capture a wider breadth of perspectives and capture less political content. In this case, a random sample detected the same themes as topic modeling, but the relative frequency from topic modeling fell in-between top-ranked and random, suggesting it is useful in qualitatively assessing relative frequency of themes. These findings have important implications for future studies to carefully consider sampling methods to uncover wider breadth of conversations about cervical cancer prevention on social media.

## Supporting information

S1 File Deidentified data. (XLSX)
